# Management of aggressive lymphoma in very elderly patients

**DOI:** 10.1002/hon.2413

**Published:** 2017-06-07

**Authors:** Catherine Thieblemont, Sophie Bernard, Thierry Molina

**Affiliations:** ^1^ Hemato‐Oncologie APHP, Hôpital Saint‐Louis Paris France; ^2^ Sorbonne Paris‐Cité Diderot University Paris France; ^3^ EA7324, Descartes University Paris France; ^4^ Anatomie‐Pathologie APHP, Hôpital Necker Paris France; ^5^ Sorbonne Paris‐Cité Descartes University Paris France

## 1 INTRODUCTION

The age defining “very elderly” patients has been determined according to the type of treatment administered, on the basis of the outcome of clinical trials. R‐CHOP can be administered until the age of 80. Beyond this, specific considerations should be taken into account, and this threshold has thus been used to define this population. Lymphoma in very elderly patients is common because approximately half of all lymphoma cases occur in patients more than 65 years old and one‐third of reported cases are aged over 75 years.

The incidence of lymphoma in older patients has increased in recent years, probably more than that of young patients, as the population aged over 60 years is continuously expanding. Although recent results showed a trend during the nineties towards stabilization of lymphoma incidence for young patients, this is not the case for older patients simply because humans are living longer and the number of older patients is consequently increasing.

Very few differences have been described between young and elderly lymphoma patients in morphology and clinical presentation. However, the outcome of elderly patients with lymphoma is worse, particularly for those with aggressive subtypes, because of the difficulties encountered during treatment and the difficulties related to the presence of other diseases, diminished organ functions, and altered drug metabolism. Until recently, very elderly patients were systematically considered too frail to receive an appropriate treatment and were thus treated with low‐dose regimens. Recent studies have concluded that the best way to improve the survival of very elderly patients with lymphoma is to choose treatment based on objective scales for the disease and the patient's general status.

## 2 INCIDENCE OF LYMPHOMA IN VERY ELDERLY PATIENTS

Life expectancy has increased dramatically over the past 50 years, with the greatest increase since the 1960s occurring between 2000 and 2015 (by 5 years). This naturally results in an increase in the number of elderly patients. Current estimates indicate that the number of people older than 65 years has more than doubled compared to 100 years ago. Individuals aged over 75 years will triple by 2030, and the group aged 85 years or older will double in the same period. This is associated with an increase in the incidence of cancer, which has been the leading cause of death, ahead of heart disease, for individuals younger than 85 years since 1999.

An increase in the incidence of lymphoma between 8% to 10% per year has been documented in Europe and the United States, particularly for patients older than 65 years, who represent half of all newly examined lymphoma patients. For the last 25 years, lymphoma incidence has increased by more than 50%, and even more than this in patients more than 60 years old, with 15 to 17 new cases a year for every 100 000 inhabitants in the United States. Several epidemiologic studies have been performed to understand this rise, in particular attempting to associate it with occupational exposures, which have changed a lot over the past 25 years. Although these analyses have not differentiated causes between young and old patients, they reveal a strong association with environmental exposure, particularly with dioxins emitted by incinerators and tobacco, with the role of pesticides being uncertain but probable, while association with sun exposure remains controversial. Recent studies linked the occurrence of lymphoma to different infectious agents, but why such infections are increasing is not known.

## 3 AGGRESSIVE LYMPHOMAS: THE MOST FREQUENT HISTOLOGICAL SUBTYPES IN VERY ELDERLY PATIENTS

All lymphoma subtypes are observed in elderly patients but with some differences compared to those encountered in younger patients. Most large epidemiologic studies done with the Working Formulation for Clinical Usage, the Revised European‐American Lymphoma classification, and the World Heath Organization classification found a higher percentage of aggressive lymphomas in the elderly. In 1997, a large study defined the differences between young and elderly patients^1^; all cases included were reviewed by 5 expert pathologists. The study revealed some differences between the 8 referral centers worldwide: elderly patients more frequently had diffuse large B‐cell lymphoma (DLBCL), peripheral T‐cell lymphoma, and lymphocytic/lymphoplasmocytic lymphoma, and less frequently anaplastic large cell lymphoma, lymphoblastic lymphoma, and Burkitt lymphoma. Smaller analyses confirmed these data particularly that for patients aged over 80, DLBCL being the most common lymphoma.^2^

## 4 VARIATIONS IN BIOLOGY OF DLBCL BETWEEN YOUNG AND VERY ELDERLY PATIENTS

Since the last decade, DLBCL biology has been increasingly understood; it is known to be highly heterogeneous, the germinal center B‐cell like/activated B‐cell like (ABC) signature being considered the major biological determinant. Remarkably, several arguments are present in the literature showing that the biology of aging has a major impact on lymphoma biology. This includes not only the germinal center B‐cell like/ABC signature, the ABC being overrepresented in patients over 80 years compared with patients aged 50 to 60 years (54% vs 33% ABC, *P* = .04), but also BCL2 expression or cytogenetic complexity, which increases with age at diagnosis.^3^, ^4^ Similarly, various genetic features, such as IRF4 translocations, 1q21, 18q21, 7p22, and 7q21 gains, as well as changes in 3q27, including gains and translocations affecting the BCL6 locus differently are significantly associated with patient age, although no cutoffs between age groups have been defined.^3^ For MYC gene rearrangement, it has been shown that the partner gene, an immunoglobulin (IG) *K*, *L* or *H* gene or not an IG, has greater prognostic value than the break itself; of importance, the median age of MYC‐IG patients is almost a decade older than MYC‐non‐IG patients (median age, 69 vs 60.5 years; *P* = .027).^5^ Recurrent somatic mutations in *CD79B, KMT2D,* and *MYD88* have been significantly correlated with age.^6^ This different biology according to age may reflect changes in the B‐cell population during aging. Another hypothesis relates to the putative pathologic specificity of DLBCL occurring in elderly patients such as the epstein barr virus (EBV)‐related DLBCL almost exclusively reported in elderly or very old patients, despite being rare in Western countries.

## 5 STAGING: SPECIAL MENTION IN VERY ELDERLY PATIENTS

Given the biological complexity of these tumors, a biopsy is an essential step in the management of aggressive lymphoma in very elderly patients. Immunochemistry is mandatory before starting treatment, while Fluorescent in situ hybridization (FISH) and genomic analyses should only be done for research purposes, as the role of these parameters is not yet clearly defined for the choice of treatment.

Relative to young patients, clinical and biological characteristics of very elderly patients with lymphoma at presentation are similar considering the main characteristics for lymphoma.^2^, ^7^ Staging to evaluate lymphoma disease should then be conducted in the same way with clinical examination, body CT scan, other examinations for clinical symptoms, blood counts, bone marrow biopsy, LDH and β2‐microglobulin measurements, human immunodeficiency virus, and hepatitis B and C virus serology. 18_FDG‐PET = 2‐deoxy‐2‐[F‐18]fluoro‐D‐glucose‐positron emission tomography (FDG‐PET)/CT is now the recommended standard for posttreatment assessment in DLBCL, irrespective of age in the last ESMO guidelines published in 2015 (Table 1).

**Table 1:**
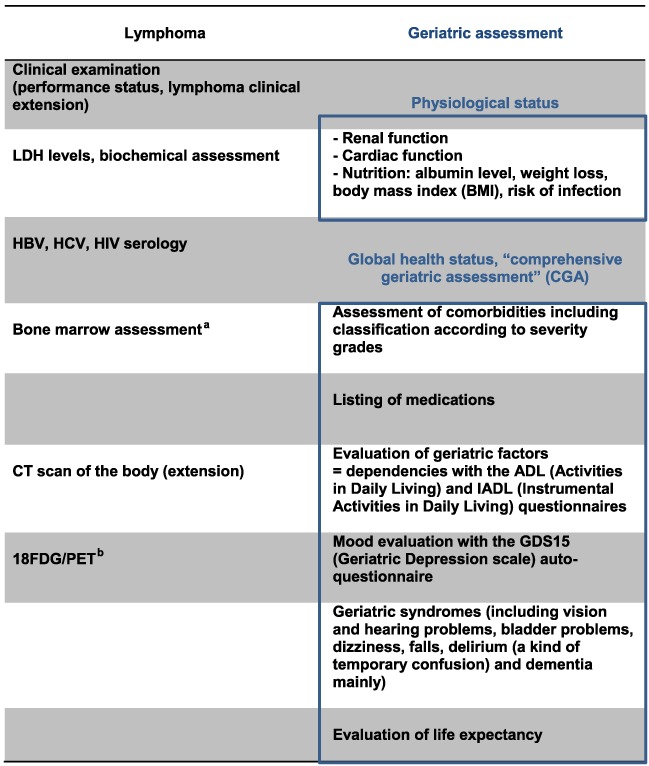
Staging in very elderly patients with aggressive lymphoma

Bone Marrow biopsy (BMB) is not anymore recommended in the staging of diffuse large B‐cell lymphoma (DLBCL) in the most recent ESMO guidelines. However, the absence of bone marrow evaluation may lead to underevaluate a specific complication associated with decreased bone marrow reserves that may lead to an increased risk of febrile neutropenia. Bone marrow puncture may be proposed if BMB is not feasible.

under evaluation.

Specific attention must be paid to comorbidities and organ dysfunctions to assess the age‐related factors. Very elderly patients frequently have comorbidities including diminished cardiac and renal function, as well as alterations in drug absorption, distribution, activation, detoxification, metabolism, and clearance, which modify the pharmacodynamics of the therapeutics. Decreases in the glomerular filtration rate and tubular reabsorption delay drug excretion, such that doses may have to be tailored to creatinine clearance. Because of decreased liver function, the metabolism of certain drugs such as cyclophosphamide or anthracyclines may be altered. However, adjustment for hepatic function was not associated with better tolerance. Hematopoietic reserve capacity may also be altered, and myelotoxicity is thus increased with standard treatment doses compared to younger patients.^8^ However, decreasing dosages because of a putative increased toxicity was proven to be associated with poorer therapeutic results. The presence of a comorbidity is associated with decreased dose intensity and decreased overall survival (OS).^9^ Details of this staging are presented in Table 1.

Evaluation of lymphoma and the geriatric assessment are performed in most centers by a hematologist. It is rare to find a concerted evaluation between a geriatrician and a hematologist due of the lack of geriatric specialists. Reliable and simple questionnaires, such as the activities in daily living and instrumental activities in daily living, are available and are adapted for routine practice to assist the hematologist to personalize the treatment strategy based on objective data.

## 6 TREATMENT

There is currently no standard treatment for very elderly patients because the most important point in this scenario is to define how to adapt treatment to the patient's specificities rather than to apply a unique regimen. However considerable progress has been made over the last decade, with retrospective and prospective studies placing median OS in this population in the range of 2.0 to 2.5 years.^7^, ^10^, ^11^ A classical approach to treat these patients has been to place them into 3 groups as defined by Balducci in 2000: fit, unfit, and frail. However, the definition of these groups for lymphoma are highly dependent on the type of treatment proposed and its expected toxicities and risks, notably febrile neutropenia, cardiopathy, number and duration of hospitalizations, neuropathy, diabetes, and osteoporosis inducing early death and functional decline.

### 6.1 The prephase

The use of prephase treatment associating oral vincristine (1 mg total dose 1 week before cycle 1 [day –7]) and oral prednisone (60 mg/m^2^ for 1 week) has been advocated, allowing a reduction in induction therapy‐associated toxicities.^10^, ^12^ Vincristine appears to be the least important component of this association, and prednisone alone may be proposed which should result in better tolerability, particularly in terms of toxicities in the first treatment cycle.

### 6.2 Chemotherapy adapted to fit patients with DLBCL

Delivery of standard dose R‐CHOP was felt to be unrealistic in the fit population, and although rituximab use was associated with decreased mortality, 1‐year OS was better when anthracycline dose intensity was <85% versus >85%, perhaps related to baseline performance status. Few prospective trials integrate a search for optimal treatment. Peyrade et al performed a multicenter single‐arm phase II trial in 150 patients aged more than 79 years old with DLBCL at diagnosis, evaluating the efficacy and safety of 6 cycles of a combination of a low‐dose CHOP chemotherapy with a standard dose of rituximab given at 3‐week intervals, (R‐miniCHOP; rituximab 375 mg/m^2^; 400 mg/m^2^ of cyclophosphamide, 25 mg/m^5^ doxorubicin, 1 mg vincristine, and 40 mg/m^2^ prednisolone for 5 days).^11^ Overall response rates were 73%, and the complete or unconfirmed complete response rate was 62%. With a median follow‐up of 20 months, median OS was 29 months and the 2‐year OS rate was 59%. The R‐miniCHOP regimen was well tolerated, allowing administration of the full planned dose in 72% of patients. The very low number of hospital admissions and deaths (12) were attributed to treatment toxicity. The most frequent toxicities were hematologic with grade 3 or 4 neutropenia in 59 patients and febrile neutropenia in 11 cases. Considering these promising results, R‐miniCHOP could be considered as the standard of care in very elderly patients with DLBCL, representing a good compromise between efficacy and safety.

### 6.3 Which regimen in patients with contraindication to anthracyclines?

To avoid cardiotoxicity associated with doxorubicin, this agent may be replaced by a nonpegylated liposomal doxorubicin (Myocet) (R‐COMP regimen). Luminari et al conducted a phase II study in 75 elderly patients (median 72 years, range 61 to 83) with newly diagnosed DLBCL and left ventricular ejection fraction (LVEF) greater than 50%. Planned treatment was 8 courses of R‐COMP.^13^ Overall response rate was 71% with 57% complete response (CR), 3‐year progression‐free survival 69%, and 3‐year OS 72% with an acceptable safety profile. R‐COMP appears to result in reduced cardiotoxicity compared to standard doxorubicin (21% cardiac event, with 4% of patients having grades 3‐4. Similarly in 2011, Corazzelli and colleagues applied a dose‐dense R‐COMP14 regimen to elderly poor‐risk patients with DLBCL. Fridrik et al conducted a phase III trial, randomizing 88 DLBCL patients to receive R‐CHOP or R‐COMP. Patients were stratified for N‐terminal pro‐brain natriuretic peptide (NT‐proBNP) serum levels and for international pronostic index (IPI) score. Only 1 patient presented a LVEF less than 50% at diagnosis and received R‐CHOP therapy at randomization. The investigators concluded that in patients with normal cardiac function at diagnosis, nonpegylated liposomal doxorubicin did not reduce cardiotoxicity, although cardiac safety signals were increased in R‐COMP compared with R‐CHOP: during treatment, LVEF measurements were less than 50% in 4.6% of patients in the R‐COMP arm, compared with 15.8% in the R‐CHOP arm (*P* < .001) and NT‐proBNP levels were less than 400 pg/mL during and at the end of treatment in 90% patients in the R‐COMP arm, but in only 66.7% in the R‐CHOP arm (*P* = .013). Efficacy was similar in the R‐COMP and R‐CHOP arms; however, this trial was not powered to detect differences in response outcome between the 2 arms.^14^

### 6.4 Chemotherapy adapted to unfit very elderly patients

Alternative regimens, investigated in very elderly patients or patients ineligible for anthracyclines, may be proposed. R‐CEOP, substituting etoposide (50 mg/m^2^ intravenously on day 1 and 100 mg/m^2^ orally on days 2 and 3 in the standard CHOP regimen) for doxorubicin was reported by Moccia and colleagues in 2009 and compared the results with a historical cohort treated with R‐CHOP, with similar 5‐year time to progression (57% vs 62%, respectively), but a lower 5‐year OS rate in patients who received R‐CEOP (49% vs 64%, *P* = .02). R‐bendamustine may also be proposed. However, results are worse, with only a 52% rate of complete response and short survival.

### 6.5 Growth factors and febrile neutropenia

In this patient population, optimal use of myeloid growth factors remains an important means of minimizing myelosuppression and subsequent infectious complications, not only to reduce morbidity and mortality but also to allow delivery of full adapted‐dose therapy which in turn impacts disease outcome. Administration should be based on ASCO and EORTC guidelines. Randomized phase III trials have confirmed the potential benefit of these agents in elderly patients. Epoetin should be used with caution in these patients with comorbidities such as hypertension.

### 6.6 Central Nervous System (CNS) prophylaxis

A recent retrospective evaluation was performed of CNS relapse in very elderly DLBCL patients aged 80 years or older treated in 2 prospective LYSA studies with miniCHOP therapy, associated with either rituximab or ofatumumab, another anti‐CD20 monoclonal antibody. This study showed a very low incidence of CNS relapse (1.8% at 2 years) despite the lack of CNS prophylaxis. This led to the conclusion that the absence of prophylaxis does not have a dramatic impact on incidence of CNS relapse and that prophylaxis can be avoided in the very elderly given the potential for the negative impact of the associated toxicities.^15^

### 6.7 Therapeutic strategies for other lymphoma subtypes

While therapeutic strategies for treating very elderly DLBCL patients are becoming clearer, very few specific propositions have been made for the treatment of other aggressive lymphoma subtypes. Burkitt lymphoma is a major problem given the poorer results obtained with classical CHOP and the near impossibility of increasing the dose intensity except in “young elderly” patients, ie, between 60 and 65 or 68 years. R‐CHOP is recommended, and if patients fail it, palliative treatment is certainly the best option. Peripheral T‐cell lymphoma is also difficult to treat because no standard has been defined for young patients. No “better” regimen than CHOP has been recommended for patients with peripheral T‐cell lymphoma.

### 6.8 Future therapeutic prospects

The R‐CHOP‐based regimen represents the gold standard chemoimmunotherapy in DLBCL, but a relevant percentage of patients still relapse or are refractory to this treatment. Better recognition of the biological basis of lymphomagenesis in very elderly patients represents a new pathway for proposing tailored treatment. Novel drugs targeting the immune system, the NFKB pathway, or the B Cell Receptor (BCR) signaling pathway, such as the immunomodulatory agent lenalidomide, inhibitors of Bruton tyrosine kinase (ibrutinib), and the proteasome inhibitor bortezomib are currently under investigation as single agents in the relapse setting and in combination with chemotherapy as first‐line treatment. Novel strategies such as the administration of maintenance therapy will also be analyzed and may offer improvement in patient outcomes.

## 7 CONCLUDING REMARKS

Age has been described as an adverse prognostic factor for survival of lymphoma patients, especially when other diseases are also present. The poorer results seen in the very elderly patients may reflect, at least partially, the use of lower doses of chemotherapeutic agents. However, once a complete remission is reached, disease‐free survival of very elderly patients does not differ from that of younger patients, emphasizing the critical importance of achieving this response outcome. As the adapted dose‐CHOP plus rituximab regimen is very well tolerated, it is currently considered standard treatment in fit patients. New immunochemotherapy agents in first‐line should help increase the complete remission rate.

## CONFLICT OF INTEREST

The authors declare no conflict of interest.
